# Beyond the Two-Clause Sentence: Acquisition of Clause Chaining in Six Languages

**DOI:** 10.3389/fpsyg.2020.01586

**Published:** 2020-08-04

**Authors:** Hannah S. Sarvasy, Soonja Choi

**Affiliations:** ^1^MARCS Institute for Brain, Behaviour and Development, Western Sydney University, Sydney, NSW, Australia; ^2^Department of Linguistics and Asian/Middle Eastern Languages, San Diego State University, San Diego, CA, United States

**Keywords:** clause chain, acquisition of complex sentence, Japanese, Korean, Ku Waru, Turkish, Nungon, Pitjantjatjara

## Abstract

Clause chains are a special type of complex sentence, found in hundreds of languages outside Western Europe, in which clauses are dependent but not embedded, and dozens of clauses can be combined into a single sentential unit. Unlike English complex sentences, clause chains’ distribution is partially predictable in that they can, most fundamentally, be linked to a particular semantic context: description of temporally sequential events or actions. This and the morphological simplicity of verb forms in clause chains may combine to accelerate their acquisition by children, relative to complex sentences in other languages. No previous cross-linguistic studies of the acquisition of complex sentences have investigated clause chaining. In this paper, we report insights from a survey of the acquisition of clause chaining in six languages of diverse stocks with child speech databases spanning 1;1 to 10 years. Overall, children acquiring clause chaining languages begin to produce 2-clause chains between around 1;11 and 2;6. An initial stage in which chains are limited to just two clauses in length is followed by a stage in which longer chains of 3–5 clauses are also produced. Children acquiring languages in which adults produce both same-subject and different-subject clause chains produce a similar mix from early on; for some languages, this involves morphological “switch-reference” marking that anticipates the identity of the subject of an upcoming clause. This survey broadens our understanding of the acquisition of complex sentences by adding new data on the acquisition timing, semantics, and reference continuity of early clause chains.

## Introduction

In hundreds of languages around the world, speakers have a third option for complex sentence formation, in addition to the well-known “subordination” and “coordination.” This third option is called a “clause chain,” and involves one or more “medial” clauses with under-inflected verbs as predicates, followed or preceded by a “final” clause with a fully inflected verb as predicate^1^ (Longacre, 1985; Dooley, 2010; Sarvasy, 2015). An example of a clause chain in Korean is in (1); throughout this paper, medial clauses are in single curly brackets and final clauses are in double curly brackets, following the convention in Sarvasy (2017): 
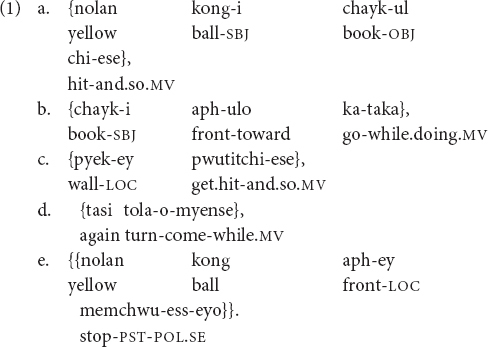


“A yellow ball hit the book and so, while the book was going forward, (the book) got hit on the wall and so, as (it) was coming back, (it) stopped in front of the yellow ball.” (Author’s Korean adult corpus)

Note in (1) that the verbal predicates of the first four “medial” clauses (1a–d) have no tense marking; only the verb in the “final” clause (1e) at the end of the chain bears tense inflection.

Clause chains are defined by three main criteria:

(a) Structure: one or more “medial” clauses (with a “medial verb” predicate that is unspecified for tense and, often, other categories) co-occur with a single “final” clause (with a “final verb” predicate that is fully specified for tense and other categories). Often, medial clauses feature rising or level pitch, whereas the final clause features a prosodic fall.
(b) Syntax: medial clauses are dependent in that they lack tense and other category specification, but they are not embedded in other clauses.
(c) Lexicon and length: there are no restrictions on which lexical verbs can occur within chains, nor the positions in which these can occur in the chain; nor are there restrictions on the length of the chain, measured in clauses, with chains of 100 clauses or more attested for some languages (Wise, 2018).

Criterion (a) rules out serial verb constructions and other multi-verb complex predicates as potential clause chains because those constructions constitute single clauses (Aikhenvald, 2018). Criterion (b) differentiates clause chaining from relative and complement clause constructions (which are embedded in other clauses) and from coordination of clauses with equal status (although the medial clauses have equal statuses to each other within the chain, they are marked as dependent, with only the final clause of the chain able to serve independently as a lone main clause). Criterion (c) distinguishes between clause chaining and adverbial clause constructions in other languages; even though adverbial clauses in languages like English can have superficially similar syntactic relations to medial clauses in clause chains, it is unnatural to combine more than about two adverbial clauses in a single English sentence.

Languages in which criteria (a–c) are satisfied can be considered “clause chaining languages.” Many of these languages exhibit two additional characteristics:

(d) The most basic function of clause chains across languages is to describe sequences of related events and actions. Although at least one study has shown that distribution of English relative and complement clauses is unpredictable based on discourse semantic content (Barker and Pederson, 2009), for clause chaining languages, there is growing evidence that clause chain use correlates with description of temporally sequential events/actions (Farr, 1999; Defina, 2020; Sarvasy, under review).
(e) In many clause chaining languages, “switch-reference” marking of medial clauses (Haiman and Munro, 1983; van Gijn and Hammond, 2016) signals in advance whether the subject of the following, as-yet unspoken, clause will be co-referential with the subject of the present clause. An example of switch-reference marking is in (2), from the Papuan language Nungon: 
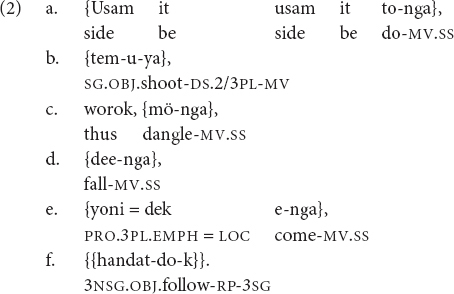

“(The group of boys) surrounding (the monster), shooting her-SWITCH, thus, dangling, falling, coming toward them, she (= the monster) pursued them.” (Author’s Nungon adult corpus)

In Nungon clause chains, medial verbs obligatorily indicate whether the subject of the following clause will differ from their own subject. If there is no anticipated difference in subject, a medial verb bears an unchanging final suffix *-nga* (*-a* after consonant-final verb roots), as in (2a, 2c, 2d, and 2e). But when (and only when) the subject of the next clause will differ from the subject of the current clause, the medial verb of the current clause bears a special subject person/number inflection before the medial verb suffix *-(y)a* (as in 2b). This inflection only indexes the subject of the current clause; no information is given in the current clause about the upcoming clause’s subject, except that it will differ. Thus, in example (2), “shoot him/her” (2b) indexes its own subject, marking that the subject of the upcoming clause (2c) will differ from the current clause’s subject. In this case, the grammatical subject switches from “a group of boys” (2a–b) to “a female monster” who was dangling from a branch (2c–f). While the subject person/number markers on the medial verb in (2b) and the final verb in (2f) indicate person and number, the identity of the two subjects is understood from narrative context.

Clause chains occur in a swath of languages across Asia, from Japanese and Korean through Mongolic, Tibeto-Burman, Turkic, and Caucasian languages. They feature in numerous languages of East Africa (especially those of Ethiopia), in some indigenous languages of North and South America, and in Melanesia. Switch-reference marking is not found in most clause chaining languages of mainland Asia, but is well-attested in the Americas and Melanesia.

In this paper, we present a first comparative sketch of child acquisition of clause chains in six languages of diverse stocks, based on the analyses of the companion papers in this Research Topic. Our motivation for this comparative study is threefold.

First, most comparative work on the acquisition/development of complex sentences has focused on clause combining structures involving coordination and subordination (e.g., relative clauses, complement clauses, and clausal coordination: Bowerman, 1979; Berman and Slobin, 1994; Diessel, 2004; Bencini and Valian, 2008; Kidd, 2011). In contrast, clause chaining – with dependent, but not embedded, clauses – does not fit easily into either category, leading Foley and Van Valin (1984) to coin a new term to describe its structure: co-subordination (and see Genetti, 2005 on the inconclusiveness of standard “tests” for syntactic relations in clause chains). Because clause chain syntax is special in not being clearly textbook subordination or coordination, our first area of investigation here is the general development of clause chaining in child speech.

Second, as pointed out in criterion (c), in clause chaining languages, it is commonplace for speakers to produce long chains including 10 or more medial clauses. Our second area of investigation concerns the developmental pattern in production of 2-clause, 3-clause, or longer chains.

Finally, switch-reference marking within clause chains means that sentence planning spans discrete clauses. If children can produce switch-reference marking accurately, this could represent advanced sentence planning abilities (Sarvasy, 2020). On the other hand, if this long-distance planning is difficult for young children, we wonder what strategies they adopt to produce clause chains without necessarily having to indicate switch-reference: do they avoid clause chaining altogether, produce reduced forms of medial verbs, or produce only same-subject clause chains? Our third area of investigation is the development of switch-reference marking and topic continuity within clause chains.

We investigated these three topics through a synthesizing analysis of this Research Topic’s acquisition studies on three Eurasian clause-chaining languages, with many millions of speakers – Japanese (Clancy, 1985, 2020^2^), Korean (Choi, 2020), and Turkish (Aksu-Koç and Slobin, 1985; Ögel-Balaban and Aksu-Koç, 2020) – as well as three under-described clause chaining languages of Papua New Guinea and Australia, each with fewer than 5000 speakers – Ku Waru (Rumsey et al., 2020), Nungon (Sarvasy, 2020), and Pitjantjatjara (Defina, 2020). Although these studies target children between 1;1 and 10 years, our emphasis in this comparative analysis is on early development, drawing primarily on the early, qualitative, longitudinal components of these studies.

The six languages share the following features: (a) verb-final constituent ordering, although Pitjantjatjara is flexible in this regard; (b) finite verbs are marked for, at least, tense or mood; (c) they are all “pro-drop” languages, with explicit personal pronouns used sparingly in discourse. The Eurasian languages (Japanese, Korean, and Turkish) differ from the Pacific languages (Ku Waru, Nungon, and Pitjantjatjara) in (a) the number of distinct semantic relations indicated by medial clauses (maximally two, sequential and simultaneous, for the Pacific group, but between four and 15 frequently used semantic relations, and up to 100 less-frequent ones, for the Eurasian languages) and (b) overt indication of switch-reference (marked through dedicated morphemes in the Pacific languages, but a covert feature of particular semantic relation types in the Eurasian languages).

With data from these six languages, we examine the following developmental aspects of clause chains: (i) general developmental patterns, (ii) chain length, and (iii) switch-reference (or topic continuity).

## Cross-Linguistic Acquisition of Clause Chaining

Children’s ages, number of participants, and size of the databases that served as the sources for the literature we consulted are in Table 1. The most relevant results for early acquisition come from the longitudinal, rather than experimental, studies; overall results from these studies are in Table 2.

**TABLE 1:** Data summary for the six clause chaining languages.

Language type	Language	Longitudinal/cross-sectional	Data collection method	Child ages	Number of children	Approximate size of corpus^a^
Eurasian	Japanese i (Clancy, 1985)	(a) Longitudinal (b) Cross-sectional	(a) Spontaneous speech recording; diary observation (b) Interviews/experimental	1;0–6;3	5 (Clancy’s) 1 (Okubo’s) + 39 children + ∼600 in cross-sectional studies^b^	^a^
	Japanese ii (Clancy, 2020)	Cross-sectional	Narrative elicitation based on still-picture cartoon and film clips	3;8–7;4	60	N/A
	Korean i (Choi, 2020)	Longitudinal	Spontaneous speech recording	1;1–5;1	5	∼37 h
	Korean ii (Choi, 2020)	Cross-sectional	Elicitation from short motion event video clips	3–10 years	60	N/A
	Turkish i (Aksu-Koç and Slobin, 1985)	(a) Longitudinal (b) Cross-sectional	(a) Spontaneous speech recording; diary observation (b) Interviews/experimental	1;0–6;4	12 adult–child interactional (Slobin and Aksu’s) + 48 cross-sectional and micro-longitudinal (Slobin and Aksu’s) + 3 longitudinal and 60 cross-sectional (Aksu’s) + 3 other longitudinal^c^	^b^
	Turkish ii (Ögel-Balaban and Aksu-Koç, 2020)	Cross-sectional	Elicitation from a picture-book	4–11 years	40	N/A
Pacific	Ku Waru (Rumsey et al., 2020)	Longitudinal	Spontaneous speech recording	1;8–4;9	4	∼40 h (32,760 child and adult utterances)
	Nungon (Sarvasy, 2020)	Longitudinal	Spontaneous speech recording	1;1–3;3	3	∼33 h (15,725 child utterances, 13,384 adult utterances)
	Pitjantjatjara (Defina, 2020)	Longitudinal	Spontaneous speech recording	0;10–10 years	28 (including 5 focus children)	4200 child utterances, 1637 adult utterances

*^a^It is hard to assess the number of hours recorded for longitudinal studies in Clancy’s (1985) and Aksu-Koç’s (Aksu-Koç and Slobin, 1985) studies, and number of hours/utterances is not applicable to the cross-sectional/experimental studies (Choi, 2020; Clancy, 2020; Ögel-Balaban and Aksu-Koç, 2020). ^b^Clancy collected 30 h of spontaneous speech data from five children, ages ranging from 1;6 to 3;6. She also consulted Okubo’s (1967) monthly recordings of her daughter’s (1 child) development from 1;0 to 6;0. For particular grammatical aspects (e.g., verbal inflections, case marking, sentence-final particles, multi-morpheme stage, negation), Clancy’s database includes longitudinal study/observation records (e.g., diary studies) of 39 children collected by different researchers. Then, for later development (3;3–6;3), there are cross-sectional/experimental studies of 300 children, which were part of a National Language Research Institute project in Japan. In addition to these, Clancy used cross-sectional data of over 300 children gathered by other researchers. ^c^12 children from 1;10 to 5;11 (adult–child interactional speech corpora, Aksu-Koç and Slobin, 1985); 48 children from 2;0 to 4;8 (cross-sectional and micro-longitudinal samples, Berkeley Crosslinguistic Acquisition Project, Slobin, 1972–1973, Aksu, 1978b; Aksu-Koç and Slobin, 1985); 3 children from 1;9 to 2;6 (longitudinal), and 60 children from 3;0 to 6;4 (experimental, investigating aspect, and modality, Aksu, 1978a; Aksu-Koç, 1988); 2 children from 1;0 to 2;0 and 1 child from 1;3 to 2;4 (monthly recordings) collected by other researchers.*

**TABLE 2:** Clause chaining early acquisition results from the longitudinal studies.

	Age range, onset of 2-clause chains	Age range, onset of 3-5-clause chains	Age range, onset of different-subject chains	First inter-clause semantics
Japanese	2;0–2;1	Unknown	Unknown	Sequential, manner
Korean	1;11–2;0 (2 children^a^)	2;0–2;4 (2 children)	1;11–2;0 (2 children)	Listing/additive, sequential, manner
Ku Waru	1;9–2;0 (2 children); 2;7–2;9 (2 children)	2;8–3;3 (only 3-clause chains)^b^	4;7 (1 child)	Idiomatic/formulaic, sequential
Nungon	2;4–2;5 (2 children)	3;1 (2 children; 1 only 3-clause chains, 1 3–5-clause chains)	2;11 (2 children)	Sequential, aspectual
Pitjantjatjara	2;8 (1 child)	5;3 (1 child)	4;1 (1 child)	Sequential, idiomatic
Turkish	2;4–2;6	5;0	Unknown	Sequential, aspectual

*^a^Because the longitudinal studies all involved staggered ages, the child counts in parentheses represent the total number of children in each study whose data were captured at this particular stage. For instance, of the 3 Nungon children in Sarvasy (2020), child 1 was studied from ages 1;1 through 2;4, child 2 from 2;1 through 3;3, and child 3 from 2;10 through 3;3. Only the two younger children’s data (child 1 and child 2) show evidence of the earliest clause chain productions (at 2;4 and 2;5), and only the two older children’s data (child 2 and child 3) show evidence of the onset of 3–5-clause chains (at 3;1 for both). ^b^Note that the Ku Waru data represent a possible exception to the generalization that 3–5-clause chains are not ordered, developmentally. 3 children produce maximally 3-clause chains from the onset of longer chains at 2;8–3;3 to the end of the study periods for them (2;11, 3;0, and 3;8). The fourth child produces maximally 3-clause chains between 3;3 and 3;9, then produces maximally 4-clause chains between 3;10 and the end of the study period (4;9). However, the numbers of long chains are very few for all children (for instance, there are just 3 utterances with 3-clause chains for 2 children).*

### General Developmental Patterns

Child production of clause chains begins around the same time in five of the six languages. Children acquiring Japanese, Korean, Ku Waru, Nungon, and Turkish show productive clause chaining of at least 2-clause chains by about age 2;6, with chaining beginning as early as 2;0 (and younger for some children in Japanese, Korean, and Ku Waru). The earliest 2-clause sentences in Nungon, observed at 2;4–2;5, are clause chains, not finite subordinate or coordinate structures. Children acquiring Pitjantjatjara, in contrast, produce 2-clause sentences that comprise juxtaposed finite verbs before producing 2-clause chains with medial verb forms. They only begin to produce 2-clause chains around age 2;8 and older. Clancy’s (2020) mixed-effects statistical model did not find that age had a significant effect on certain properties of clause chains in Japanese, such as chain length, implying that by 4 years, Japanese children’s clause chain productions are relatively adult-like. For Korean and Turkish, at least, there is further development around 10 years of age that brings clause chain productions closer to the adult grammar (in terms of medial verb forms and chain lengths). Because the other four studies did not include children of over 8 years, it remains to be seen whether this is also the case in the other languages.

For all the languages, children show early production of clause chains involving sequential actions/events (Table 2). Three languages (Korean, Ku Waru, Turkish) enable speakers to specify, through the particular medial verb suffix, whether actions denoted by adjacent clauses occur simultaneously or sequentially. In these languages, production of chains denoting simultaneous actions tends to develop later. The Eurasian languages have from four (Turkish) to at least five (Japanese; Iwasaki, 2002, p. 60) to 100 (Korean; see Sohn, 2009) distinct medial verb suffixes to indicate specific inter-clausal semantic relations (see example 1). For Korean, the forms indicating sequentiality, cause, and manner are among the earliest to be used by children, from 2;0. In contrast, the Pacific languages have the means to express maximally just two inter-clausal semantic relations: temporal sequentiality and simultaneity (in Ku Waru). In Nungon and Pitjantjatjara, there is a single medial verb form with general sequential temporal semantics. Nungon and Pitjantjatjara medial clauses can have extended semantic interpretations, including aspectual, conditional, and causal, but these must be deduced from context because they are not indicated formally (see example 2).

In sum, these data taken together suggest that at 2;0–2;6, children are ready to join two ideas in a clause chain, regardless of language-specific morphology, and that by 4 years, they have acquired the basic structure of clause chains in the target language (see similar findings by Berman and Slobin, 1994, for other types of complex sentences). The developmental order of semantic functions can be explained, at least in part, by the degree of concreteness of the events/states being chained.

### Clause Chain Length: From 2-Clause to Longer Chains

Both language and individual differences impact the ages at which children produce chains of three or more clauses. For all languages, children begin by producing 2-clause chains before producing longer ones. After this initial stage, children vary – within and across languages – in the age at which they produce longer chains (of ≥3 clauses). Children also vary as to whether their first longer chains are limited to just three clauses for an extended period, or immediately range from three to five clauses, but the overall picture from the data is that there is no consistent ordering pattern in acquisition of 3-clause, 4-clause, and 5-clause chains. For example, the first clause chains of one Korean child were 2-clause chains (2;0 through 2;3). However, in the single session at 2;4, he produced one 4-clause chain and one 5-clause chain (and no 3-clause chains), among a number of 2-clause chains.

Onset ages for production of longer chains (≥3 clauses) vary widely, between 2;0 and 5;3. The children acquiring Korean began producing chains of 3–5 clauses the earliest: one child at 2;0, one month after beginning to produce 2-clause chains, and another at 2;4, in the fourth month after beginning to produce 2-clause chains. In Ku Waru, 3-clause chains are attested only from 2;8, and in Nungon from 3;1 (when one child begins to produce 3–5-clause chains, and the other begins a 3-month stage of maximally 3-clause chain production). In Japanese, one child already produced a 20-clause chain at 3;10, while narrating a story based on a nine-panel cartoon; as noted above, clause chain use in some languages has been shown to correlate with discourse genre, with high frequency and possibly greatest length in narratives (Farr, 1999; Defina, 2020; Sarvasy, under review).

Children acquiring Pitjantjatjara and Turkish show the slowest developmental trajectories of the six languages in terms of chain length. In Pitjantjatjara, young children mostly produce 2-clause chains, with the first 3-clause chain attested in the speech of a child of 5;3. Although the small sample size for this study could have impacted this result, clause chains in adult child-directed Pitjantjatjara speech are very infrequent, featuring in only 3% of utterances (Defina, 2020), and short: rarely more than three clauses in length. In Turkish, children still produced only 2-clause chains at age 4, with 3- and 4-clause chains attested from 5 years of age. Even adult narratives in the Turkish study contained maximally 4-clause-long chains, in contrast to adult chains in Japanese, Korean, Ku Waru, and Nungon.

Differences between these groups cannot be attributed to relative morphological or semantic complexity of the verb forms involved in clause chaining: in each language, there is at least one “same-subject” (see next section) medial verb form with maximally general, temporally sequential meaning, formed with a monosyllabic suffix after the verb root (Japanese *-te*, Korean *-ko*, Nungon -*nga*/*-a*, Pitjantjatjara *-la/-ra*, Turkish *-Ip*, and Ku Waru various forms with subject inflection). In only Ku Waru does this general form also incorporate subject person/number, and then the distinctions made are still fewer than in final verbs.

Diverse study designs for the different languages could have affected clause chain length, by both delimiting discourse genre and shaping how narratives were subdivided into sentential units. In the longitudinal Korean, Ku Waru, and Nungon studies, parents engaged the target children in play and conversation in an indoor setting. The Japanese and Turkish studies targeting children of 3;8–4;0 and older both elicited narratives from participants, but in different ways. The Japanese study involved: (a) story-telling while viewing a series of events depicted in nine panels laid out horizontally in front of the narrator, or (b) free re-telling of stories after viewing videos. In contrast, Turkish study participants narrated a picture-book while viewing the book, page by page: this pacing could have limited clause chain length in the results for Turkish (evinced in the limited and relatively uniform length of even adult Turkish clause chains, in contrast to the greatly varying lengths produced by Japanese adults). The Pitjantjatjara study was the most naturalistic: target children wore audio recorders as they moved freely in an outdoor location among a few family members. This design could have limited child and adult clause chain length by limiting occasions to tell stories.

Alternatively (especially because Clancy’s, 2020 statistical model found no difference in Japanese clause chain length between the nine-panel cartoon task and the video re-telling task), it could be the case that study design is less important than general preferences of each speech community for clause chain length; Alan Rumsey (p.c., 2020) reports that clause chains of 10 or more clauses are rare in even narrative adult Ku Waru, in contrast to adult Nungon, Japanese, and Korean (average clause chain length in adult narratives is unknown for Turkish and Pitjantjatjara).

### Switch-Reference and Topic Continuity Within Chains: Is Co-referentiality Preferred Early?

Developmental patterns for switch-reference and topic continuity in clause chaining languages are highly language-specific. Co-referentiality of the subjects of adjacent chained clauses (“same-subject”) or non-co-referentiality (“different-subject”) is obligatorily morphologically indicated through switch-reference marking on medial verbs in Ku Waru and Nungon (as in example 2); in Pitjantjatjara, different-subject is indicated through a free particle. In Japanese, Korean, and Turkish, switch-reference is not indicated morphologically, but medial verb morphemes indicating particular inter-clausal semantic relations tend to be associated with same-subject or different-subject contexts, or allow either. In Japanese and Korean, for example, the medial verb suffix denoting “simultaneity” predominantly favors cross-clause subject maintenance, whereas the suffix denoting “additive” allows for either subject maintenance or a change in subject. In Turkish, the medial verb forms that can function in either same-subject or different-subject contexts are accompanied by null pronouns in same-subject contexts and explicit pronouns in different-subject contexts.

The early 2-clause chains produced by children acquiring Ku Waru, Nungon, and Pitjantjatjara all involve cross-clause subject maintenance. One Nungon child produced only same-subject chains from 2;4 through 2;10, after which she produced her first different-subject chains. However, that child produced very few 2-clause chains at all in that period, so sample size could be a factor here. Ku Waru children show a pronounced delay in production of different-subject chains, with the first token produced at 4;7 by the oldest child in that study; this is likely related to a strong preference in that language for subject maintenance throughout chains (see below). Children acquiring Pitjantjatjara also produce different-subject chains after a sizable delay, by around 4;1.

For Korean, children’s earliest 2-clause chains are a mix of same- and different-subject chains. The earliest clause chains produced by Japanese children employ the *-te* medial verb form in same-subject contexts (Clancy, 1985), and it is unclear when children begin to produce different-subject chains in Japanese. Previous work on Turkish acquisition reported that children’s first medial verb forms are a mix of those permitting only subject maintenance, and those allowing for either subject maintenance or subject switch (Slobin, 1995, pp. 350–351).

Overall, the data on early productions of switch-reference in clause chains indicate a strong effect of language-specific input, rather than constants of cognitive constraints. The marked developmental delay in production of different-subject clause chains by children acquiring Ku Waru and Pitjantjatjara can be attributed in large part to the very low proportions of different-subject clause chains in child-directed adult speech in these languages. In most Ku Waru transcripts, 100% of adult clause chains involve a single subject that is maintained throughout the chain (Rumsey et al., 2020). Pitjantjatjara is similar; all but one adult clause chain token in the sample were same-subject (Defina, 2020). On the other hand, Korean children produce same- and different-subject chains from early on, as the target language provides both. Nungon data indicate something similar: at the age when children’s clause chain production increases (around 2;11), they produce a mix of same- and different-subject clause chains, just as Nungon adults do consistently.

## Discussion

This comparative study of the acquisition of clause chaining across six typologically diverse languages contributes several insights to research on the acquisition of complex sentence structures.

### Early Semantically Complex, Error-Free Production

In five of the six languages, 2-clause chain production begins between 2;0 and 2;6. This timing is similar to the early production of complement clauses in French (Dye, 2005) and Polish (Smoczyńska, 1985), relative clause-like structures in English (Diessel, 2004) and French (Dye, 2005), and adverbial and coordinate clauses in child English (Clark, 2003; Diessel, 2004). However, early clause chains differ from early clause combinations in other languages in two ways. First, Diessel (2004) proposed that children’s earliest complement and relative clauses in English involve a single semantic proposition. This is clearly not the case with children’s early clause chains for at least some of the languages studied here (Japanese, Korean, Nungon, Turkish), where early chains describe two temporally sequential distinct concrete actions or events. Second, early clause chains are morphologically well-formed, whereas the early adverbial and coordinate clauses in English tend to be morphologically lacking (usually omitting conjunctions).

### Quick Progression in Number of Clauses Combined Into Sentences

Here, study of clause chaining adds a further dimension – number of clauses combined – to the existing literature on complex sentence development (summarized in Lust et al., 2015). For all six languages, children begin by producing only 2-clause chains. It takes at least a month and in most cases several months, or even several years, for children to produce longer chains (usually, in the first instance, of 3–5 clauses). Beyond the 2-clause stage, chain length is generally open, not limited to three clauses. This transition can be likened to the well-known developmental phenomenon in which children transition from the 2-word to the multi-word stage, in which children combine not just three but several words at a time.^3^

### Early Ability to Indicate Relations Across Linked Clauses

Sarvasy (2020) suggested that switch-reference marking in clause chains could pose a cognitive hurdle for children, in that it apparently requires speakers to plan chains at least two clauses at a time (to be able to mark in advance whether the subject of the upcoming clause will be the same or different). Sarvasy (2020) proposed that children had three hypothetical options to mitigate the cognitive demands of switch-reference marking: (a) produce other types of complex sentences (which do not require such cross-clause reference tracking) before ever producing clause chains; (b) use only same-subject chains, to avoid having to track subjects; or (c) use morphologically reduced medial verbs to avoid either same-subject or different-subject marking within clause chains. Sarvasy (2020) showed that children acquiring Nungon do not pursue strategy (a) or (c), whereas there is limited evidence that one child uses strategy (b) for about 5 months (2;5–2;10). The use of same-subject chains for a much more extended period by Ku Waru and Pitjantjatjara children probably stems from distributions in the ambient language. Because some medial verb forms in Japanese, Korean, and Turkish allow for either subject maintenance or switch across clauses, children acquiring these languages are potentially spared the challenge of advance planning because they do not have to commit in advance to subject maintenance or switch.

## Conclusion

In sum, most children, regardless of language, show the ability to produce complex 2-clause sentences (or precursors of these) from around their second birthday. However, children acquiring some clause chaining languages seem to do this with more semantic and lexical flexibility than attested for early subordination in other languages; well-formed morphology, unlike early coordination in other languages; early expansion from two to more clauses; and an early ability to plan across clauses, flagging information about the subject of the next clause in advance. In our view, the features of clause chaining that facilitate acquisition include medial verbs’ formal and semantic simplicity, their consistent occurrence in the prosodically salient clause-final position (*vid.* Slobin, 1973), and the predictable use of clause chains by adults in semantic contexts involving temporally sequential events/actions, unlike complex syntax in languages like English (Barker and Pederson, 2009).

Of the source studies, only one (Sarvasy, 2020) compared clause chain development with that of coordination and subordination in a single language. Sarvasy (2020) showed that two children acquiring Nungon show marked upticks in production of all three complex sentence types around age 2;11, and from that point on, Nungon clause chain counts greatly outpaced counts of subordinate and coordinate sentences in child speech. It remains to be seen whether other “clause chaining languages” show similar patterns, with clause chains preferred over other complex sentence types in early child productions.

We further suggest that the clause chaining/non-clause chaining language distinction be applied to re-evaluate earlier results in studies of descriptions of motion events. Earlier work has shown that children learning particular languages (such as Korean) express more semantic components of motion (Path, Manner, Cause) per utterance unit than children learning other languages (e.g., English, French) (Choi, 2014). This cross-linguistic difference was typically discussed in terms of Talmy’s typology of lexicalization of motion events, i.e., verb- versus satellite-framed languages (Talmy, 1985). However, the use of clause chains in Korean could serve as an alternative explanation: children learning a clause chaining language can link several clauses in one utterance, enabling them to express several components of a motion event, whereas children learning a non-clause chaining language are restricted (by the input language) to linking maximally about two clauses (e.g., one main and one subordinate clause).

Finally, the studies compared in this paper are diverse in many respects, beginning with the extreme differences between the speech communities’ lifestyles, from the industrialized, mid-to-high-socioeconomic-status children in the Eurasian language studies to the remote, traditional small communities in the Pacific language studies. The studies further differed in number of target children, children’s ages, elicitation task types, or naturalistic data collection methods. Systematic investigation of children’s production of clause chains in a more controlled manner – targeting, for instance, clause chain distribution across different discourse genres in child speech – remains on the horizon.

## Data Availability Statement

This study does not involve any direct research but only cites published data. Requests to access the data analyzed in this article should thus be directed to the relevant researchers for each of the six language studies.

## Ethics Statement

Studies cited in this paper involved Ethics approvals from relevant bodies, which are noted in those publications.

## Author Contributions

HS and SC contributed to the writing and data analysis. Both authors contributed to the article and approved the submitted version.

## Conflict of Interest

The authors declare that the research was conducted in the absence of any commercial or financial relationships that could be construed as a potential conflict of interest.

## Footnotes

1 It is conventional in much literature on clause chaining to use “final” to describe the verb forms that are fully specified for tense and other categories, as well as the clauses that include such verbs. This term originated in literature on languages in which the clause with the fully-specified verbal predicate comes last in the chain. Because all six languages discussed here belong to this category, the term is used here.

2 All data from the six target languages comes from these sources, except where otherwise noted. Note that Clancy (1985) covers the age range between approximately 2;0 and 3;8, where Clancy (2020) begins; similarly, Aksu-Koç and Slobin (1985) covers the age range between approximately 2;4 and 4;0, where Ögel-Balaban and Aksu-Koç (2020) begins.

3 More speculatively, one might compare these stages in acquisition to the literature on infants’/toddlers’ comprehension of number concepts, showing that the difference between 1 and 2 is salient, but between 3 and a few more is not (Xu et al., 2005; Condry and Spelke, 2008).

## Abbreviations

1SG, etc.: person/number
DS: different-subject
EMPH: emphatic
LOC: locative
MV: medial verb
NSG: non-singular ( > 1)
OBJ: object
PL: plural ( > 2)
POL: polite
PRO: pronoun
PST: past tense
RP: remote past tense
SBJ: subject
SE: sentence-ending
SG: singular
SS: same-subject.
